# Fault Diagnosis for Rolling Element Bearings Based on Feature Space Reconstruction and Multiscale Permutation Entropy

**DOI:** 10.3390/e21050519

**Published:** 2019-05-23

**Authors:** Weibo Zhang, Jianzhong Zhou

**Affiliations:** School of Hydropower & Information Engineering, Huazhong University of Science and Technology, Wuhan 430074, China

**Keywords:** fault diagnosis, rolling element bearings, ensemble empirical mode decomposition, feature space reconstruction, multiscale permutation entropy

## Abstract

Aimed at distinguishing different fault categories of severity of rolling bearings, a novel method based on feature space reconstruction and multiscale permutation entropy is proposed in the study. Firstly, the ensemble empirical mode decomposition algorithm (EEMD) was employed to adaptively decompose the vibration signal into multiple intrinsic mode functions (IMFs), and the representative IMFs which contained rich fault information were selected to reconstruct a feature vector space. Secondly, the multiscale permutation entropy (MPE) was used to calculate the complexity of reconstructed feature space. Finally, the value of multiscale permutation entropy was presented to a support vector machine for fault classification. The proposed diagnostic algorithm was applied to three groups of rolling bearing experiments. The experimental results indicate that the proposed method has better classification performance and robustness than other traditional methods.

## 1. Introduction

Bearings are a critical mechanical part of rotating machines, and their health condition influences the running safety of the corresponding rotor and even the whole mechanical system. It is found that many failures of rotating machines are caused by faults of rolling bearings. Bearing fault diagnosis attracted considerable attention in previous studies. Substantial research was developed in the areas of signal analysis, feature extraction, and fault diagnosis in recent years.

Bearing failures can be caused by a considerable number of factors, such as strikes, corrosions, friction, loading, and design defects. The dynamic behavior of rolling bearings tends to be complicated and flexible, and the vibration signal exhibits nonlinear and non-stationary characteristics. Many methods for fault diagnosis were developed, such as model-based methods [1,2], observer-based methods [3,4], and data-driven methods [5,6,7]. Analysis of vibration signal is a key technique for bearing fault diagnosis. Traditional vibration signal analysis methods contain time statistics and Fourier transform. Time domain statistics, including indicators of peak amplitude, the root mean square, crest factor, kurtosis [8,9,10], and Fourier transforms, such as Fourier spectra and cepstrum analysis [11,12], were shown to be suitable for feature extraction. Nevertheless, they do not perform well enough when dealing with nonlinear and non-stationary signals. Thus, typical time–frequency analysis methods such as wavelet transform (WT), variational mode decomposition (VMD), and empirical mode decomposition (EMD) were developed. Wavelet transform shows great advantages in de-noising, fine resolution, and signal analysis [13]. However, its mathematical model and parameters are hard to determine. VMD has a more robust capability than WT, and it is used in many fields [14,15]. EMD [16], as a time–frequency analysis approach, was developed and is widely applied to feature extraction and fault diagnosis of rotating machinery [17,18,19]. It can decompose the signal into a set of complete components named intrinsic mode functions (IMFs) based on local characteristics. Nevertheless, EMD is engaged in a major drawback called mode mixing. It is denoted as a single IMF consisting of components with a wide range of different scales, or similar components existing in different IMFs [20,21]. Ensemble empirical mode decomposition (EEMD) is an improved version of EMD. It was proposed to alleviate mode mixing in EMD by introducing white noise to the investigated signal. As it greatly improves the quality of IMFs in EMD, it is widely applied in signal analysis and fault diagnosis [22,23,24]. In addition, due to the variable operating conditions and complex causes of faults, the vibration signal is usually characterized by nonlinearity. Single time–frequency analysis techniques have limitations in extracting nonlinear characteristics that are masked in the vibration signals and, thus, cannot effectively reflect inherent diagnostic information. 

The development of nonlinear parameter estimation techniques made entropy-based methods an important approach for diagnosis. Approximate entropy (ApEn) [25], as a complexity measure tool, was utilized to detect structural defect faults in rolling bearings. However, this method is weak in dealing with short datasets and leads to inconsistent results [26]. To overcome the drawbacks of ApEn, sample entropy (SampEn) was introduced by Richman and Moorman [27]. To improve the performance of the conventional complexity calculation approach based on a single scale, Costa et al [28] proposed an enhanced method called multiscale sample entropy (MSE). MSE, which is based on SampEn, was utilized to measure the regularity of complex data from multiscales. However, the SampEn-based method provides a bad estimation in view of the fact that the existence of outliers and artefacts changes the time-series standard deviation, as well as the similarity criterion [29]. Furthermore, it will bring a larger computational cost. Valencia et al. [30] proposed refined MSE to overcome two shortcomings of MSE and confirmed that additional information can be obtained by measuring complexity of the temporal scale. Azami et al. [31] introduced refined multiscale fuzzy entropy based on standard deviation to solve the issue of undefined MSE values when dealing with short time series. Yeh et al. [32] introduced the generalized multiscale Lempel–Ziv complexity method to research complexity of nonlinear systems over multiple time scales. In recent years, permutation entropy (PE) was proposed by Bandt [33,34] to detect the complexity of mechanical systems. PE has the characteristics of a simple concept and fast calculation, which is very suitable for detecting qualitative and quantitative dynamical changes [35]. Xiao [36] utilized the intrinsic mode permutation entropy (IMPE) approach based on PE to extract features in bearings. However, the PE method has limited performance in observing the dynamic changes of vibration signals only according to a single scale. To overcoming this shortcoming, a new method named multiscale permutation entropy (MPE) was proposed by Aziz et al. [37]. Compared with PE method, MPE provides more details on the dynamic behavior of the vibration signal over multiple time scales. Furthermore, it attracted increasing attention from researchers in the area of fault diagnosis. Wu [38] used the MPE approach to extract fault information from failed bearings. Vakharia et al. [39] employed MPE to pick optimal wavelets served for feature extraction. Zheng and Cheng [40] extracted the nonlinear fault characteristics from the bearing vibration signal based on the MPE method. However, the MPE has limitations when differentiating between distinct patterns of a certain motif and the sensitivity of patterns close to the noise floor [41]. Vibration signals tend to be more complex and irregular when rolling bearings are operated under harsh conditions. Thus, it is hard to recognize multiple fault statuses of bearings using the MPE method directly.

In this study, a novel method for bearing fault diagnosis is developed using EEMD-based feature space reconstruction and the MPE technique. The feature space reconstruction includes two parts: the acquisition of IMFs by EEMD and the reconstruction based on IMFs. Firstly, the bearing vibration signals are decomposed by EEMD. Secondly, the IMFs that contain abundant fault information are selected based on criteria of the energy measure to reconstruct the feature space. Finally, the complexity of the new reconstructed feature space is calculated by MPE over multiple time scales, and the results are presented to a multi-fault classifier for fault classification. As support vector machines (SVMs) have excellent performance for smaller sample data and fast training [42,43,44], an SVM was applied to identify different fault categories and degrees in this paper.

The remainder of the paper is organized as follows: Section 2 presents the EEMD-based feature space reconstruction. Section 3 shows definitions of PE and MPE. Section 4 describes the proposed method in detail. Section 5 focuses on the experimental validation of the proposed method. Finally, conclusions are given in Section 6.

## 2. EEMD-Based Feature Space Reconstruction 

### 2.1. A Brief Overview of EMD and EEMD

The EMD can decompose a signal into a sum of IMFs, each of which is a function of a time series that can reflect inherent imbedded structures [16].

Given a signal *x(t)*, the general process of the EMD algorithm is as follows:

(1) Identify the whole local extrema points of *x(t)*, and fit envelopes of *x(t)* according to the corresponding extrema points using a cubic spline function.

(2) Calculate the local means *m(t)* of envelopes, and subtract the local means from *x(t)*; then, obtain a new time series *h(t)*, where *h(t)* = *x(t)* − *m(t)*. Repeat this progress until the mean values of envelopes tend to zero. The first IMF *c*_1_ can be obtained as the highest frequency of the time series.

(3) Subtract the IMF from the original signals; a new time series is obtained and served as the input in step 1. Then, repeat the above 2 steps until the last time series is a monotonic function or small enough.

Finally, we can obtain

(1) $$x(t)=\sum_{i=1}^{n}c_{i}(t)+r_{i}(t),$$

where *c_i_(t)* denotes the *i*-th IMF, and *r_i_(t)* denotes the residue of the signal.

However, there exists a significant problem called mode mixing in the EMD method, which is defined as a single IMF including different oscillation scales or a similar scale presenting different IMFs [23]. The possible factors causing mode mixing include intermittency of the data, impulse interference, noise pollution, etc. When mode mixing occurs, the physical meaning of IMFs will make no sense. 

To alleviate mode mixing in EMD, Huang [20] introduced the EEMD method by adding white noise to the analyzed data. Thus, united distributed reference scales can be provided throughout the whole time–frequency space. The signals were able to spontaneously decompose into the sums of proper components. The algorithm of EEMD can be briefly summarized as follows:

(1) Add a white noise series *n_i_(t)* to the original signal *x(t)* (repeated M realizations in this work), where *n_i_(t)* represents the *i*-th added zero-mean noise. Then, *x_i_(t)* = *x(t)* + *n_i_(t)*.

(2) Decompose the noise-added signal *x_i_(t)* into *K* IMFs *c_ij_(t)* (*i* = 1, 2, …, *K*) by EMD, where *c_ij_(t)* presents the *j*-th IMF in the *i*-th realization.

(3) Compute the ensemble mean as follows:

(2) $$c_{j}(t)=\frac{1}{M}\sum_{i=1}^{M}c_{ij}(t).$$

In EEMD, two important parameters need to be specified: the amplitude of added white noise and the ensemble number. These two parameters directly influence the performance of EEMD. A smaller amplitude will result in lower decomposition errors. However, if too small, it will have little effect on solving mode mixing. Meanwhile, the added white noise hardly influences the performance of decomposition when the ensemble number is big enough. In general, it is recommended that the amplitude of added noise should be 0.2 standard deviations. An ensemble number of a few hundred will result in an exact result [20,21,22].

### 2.2. Feature Space Reconstruction Based on EEMD (FSRE)

The EEMD technique performs as an adaptive data-driven technique which decomposes the signal into multiple IMFs representing its inherent oscillatory modes. Each IMF generated by EEMD can be viewed as a characteristic “feature” embedded within the signal. Due to large noise or other nonlinear factors, parts of IMFs such as noise and residue components are considered as interference terms or false components, which are useless for feature extraction. The energy content is a direct measure for the strength of a signal. It is used as a key indicator for IMF selection in terms of extracting features [18]. In this section, a feature space reconstruction approach is presented to extract fault features. 

The method includes two steps. Firstly, the EEMD method is employed to decompose the original signal into a set of IMFs that represent the oscillating components of different frequency bands. In the second step, the most representative IMFs that contain rich fault information are selected to construct the feature space according to the criteria of the energy measure. The details are presented below.

Given any signal *x(t)*, we can obtain $x(t)=\sum_{i=1}^{n}c_{i}(t)+r(t)$ according to Equation (1) using the EEMD method. Each of these IMFs representing a specific range of frequency components contains a certain amount of information. It is noted that the IMFs are independent and orthogonal with respect to each other [16]; the feature space can be reconstructed by multiplying IMFs over each scale with the relevance factor $\varphi_{i}(t)$, and summarizing them as follows:

(3) $$\hat{x}(t)=\sum_{i=1}^{n}c_{i}(t)\varphi_{i}(t)+r(t),$$

where $\hat{x}(t)$ is the reconstructed feature space, and $\varphi_{i}(t)$ is the relevance factor.

The value of the correlation factor $\varphi_{i}(t)$ is related to the importance of the information contained in the IMFs. It is measured according to the criteria of the energy, which is expressed as

(4) $$\varphi_{i}(t)=\left\{ \begin{array}{ll} 1 & if\;E\left(c_{i}\right)\geq Threshold\\ 0 & otherwise \end{array}\right.,$$

where $E\left(c_{i}\right)$ denotes the energy contained in the *i*-th IMF.

The threshold is determined according to the following criteria: (1) ensure the sum energy of the selected IMFs exceeds 0.95 times the total energy; (2) eliminate insignificant or irrelevant components which contribute less to fault diagnosis (such as the residue of the signal by EEMD). Let *Threshold* = *E(c_j_)*, when *E(c_j_)*/*E*(*c_j_*_+ 1_) > *§* and $\sum_{j=1}^{j}c_{j}/\sum_{j=1}^{n-1}c_{j}>0.95$, where *j* = 1, 2, 3, …, *n* − 1, *E*(*c*_1_) > *E*(*c*_2_) >…> *E*(*c_n_*_− 1_), and *§* is the closeness measure of energy between adjacent components, which is usually set as 10.

### 2.3. Analysis of Simulating Bearing Fault Signals

Generally, most of the faults in bearings are often caused by dimensional defects. A local fault may produce periodic impacts. When the defect is subjected to a periodic pulsating load, a cyclically varying contact stress is generated, and it is reflected in the resonance modes across the spectrum of the signal. Taking the fault in the outer race as an example, it can be described by Equation (5).

(5) $$s(t)=\left[1+A\cos(2\pi f_{o}t)\right]\cos(2\pi f_{r}t)+N(t),$$

where *S*(*t*) denotes the outer race fault, *f_o_* is the characteristic frequency of the outer race, *f_r_* is the modulation frequency, and *N(t)* is the white noise. Here, we set *A* = 1, *f_o_* = 105 Hz, *f_r_* = 400 Hz, and the signal-to-noise ratio (SNR) of added white noise to −9.5 dB.

**Figure 1:**
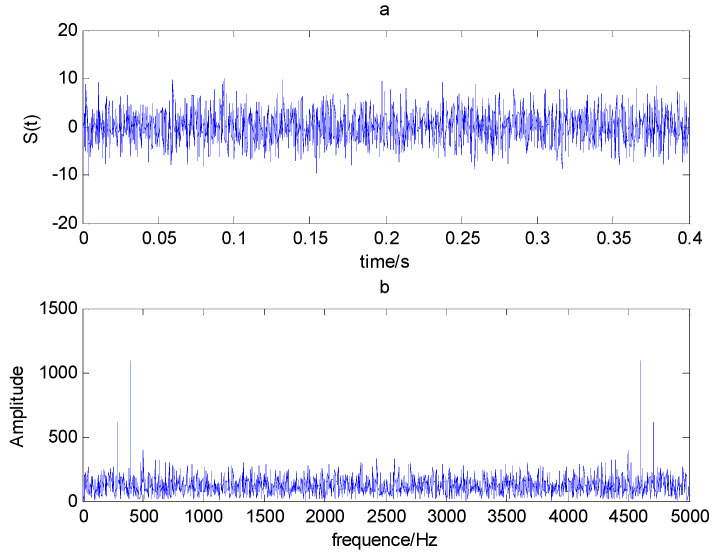
(**a**) Time domain waveform; (**b**) frequency spectrogram.

**Figure 2:**
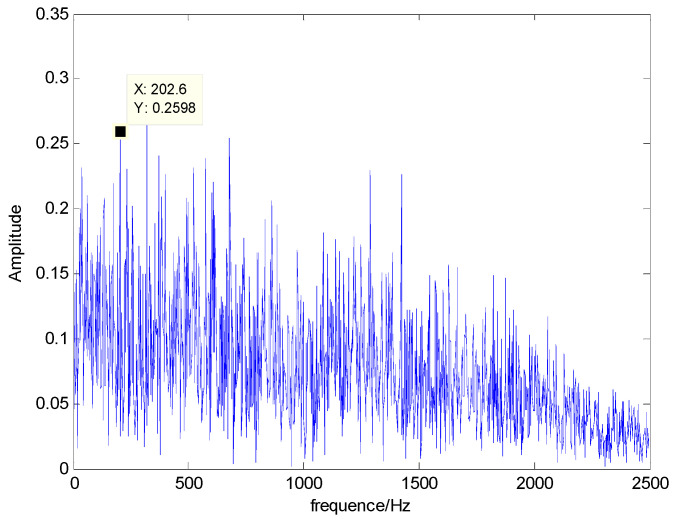
Envelope spectrum of *S(t)*.

**Figure 3:**
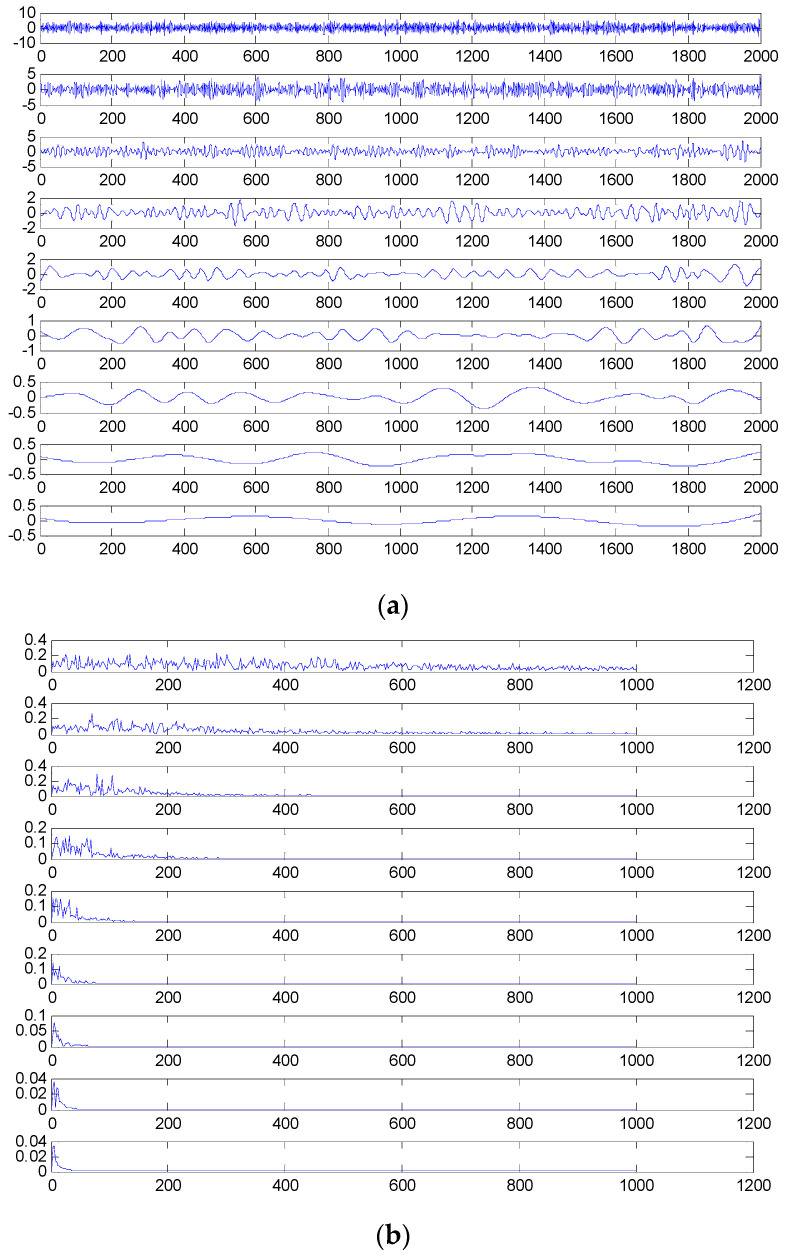
(**a**) The decomposed results of *S(t)* using ensemble empirical mode decomposition (EEMD); (**b**) envelope spectrum of intrinsic mode functions (IMFs).

**Figure 4:**
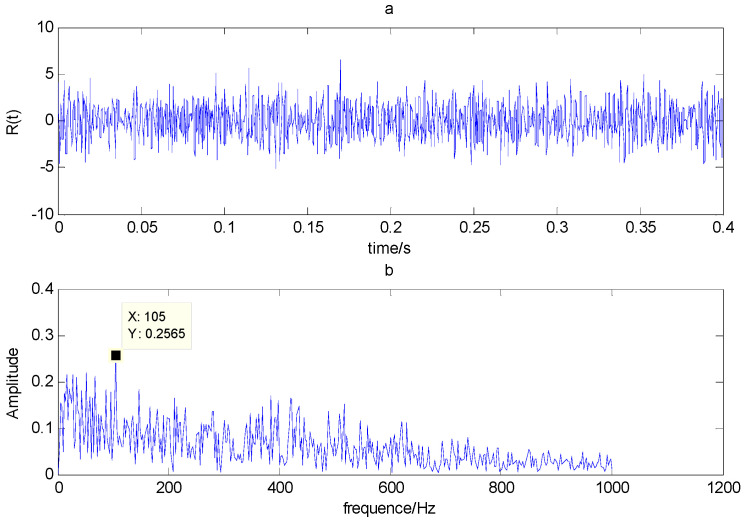
(**a**) Time domain waveform of *R(t)*; (**b**) envelope spectrum of *R(t)*.

The time domain and frequency waveform of the simulated signal are presented in Figure 1. It can be clearly seen that there are periodic impacts and frequency modulations in the signal. Due to the noise corruption, no apparent frequency components of the outer race defect could be identified from Figure 1. The envelope demodulation technique is a good method when dealing with the modulation problem [19]; however, the characteristic frequency of 105 Hz is not clearly differentiated from other components in the envelope spectrum of the original signal, as shown in Figure 2. Figure 3 presents the IMFs of the simulation signal obtained by EEMD and its corresponding envelope spectrum. In Figure 4, the waveform and envelope spectrum of reconstruction result *R(t)* is shown. From the envelope spectrum of Figure 4b, the characteristic frequency of 105 Hz can be clearly captured, which can be used to roughly identify the outer race fault. The complexity of feature space reconstruction is further analyzed to distinguish faults with different severities and categories in the next section.

## 3. Permutation Entropy and Multiscale Permutation Entropy

### 3.1. Permutation Entropy

Given a time series {*x*(*i*), *i* = 1, 2, …, *N*}, the permutation entropy can be described as follows [33,34]:

(1) Reconstruct the phase space of time series *x*(*i*); then, the reconstructed matrix *A* can be obtained as follows:

(6) $$A=\left[\begin{array}{l} x(1),x(1+r),\cdots ,x(1+(m-1)r)\\ \vdots \\ x(j),x(j+r),\cdots ,x(j+(m-1)r)\\ \vdots \\ x(n-(m-1)r),x(n-(m-1)r+r),\cdots ,x(n-(m-1)r+(m-1)r) \end{array}\right],$$

where *m* is the embedded dimension, and *r* is the time delay. 

(2) Sort the reconstructed components and obtain symbol permutations; the *j*-th row of matrix *A* is obtained follows:

(7) $$X(i)=\left\{x(i+(j_{1}-1)r)\leq x(i+(j_{2}-1)r)\leq \cdots \leq x(i+(j_{m}-1)r)\right\}.$$

If *x*(*i* + (*j*_1_ − 1)*r*) = *x*(*i* + (*j*_2_ − 1)*r*), their original positions can be sorted according to the index j*. Then, any time series *X*(*i*) can be transformed into a collection of symbols as

(8) $$S(k)=(j_{1},j_{2},\cdots ,j_{m}),$$

where *S*(*k*) contributes the *k*-th permutation among the *m*! different symbols. 

(3) Define permutation entropy (PE); if the probability of each symbol sequence is identified as *P*_1_, *P_2_*, …, *P_l_*, consequently, the PE can be defined as the Shannon entropy,

(9) $$H_{p}(m)=-\sum_{j=1}^{l}P_{j}\ln P_{j},$$

and it can be normalized as

(10) $$H_{p}=\frac{H_{p}(m)}{\ln(m!)},$$

where 0 ≤ *H_p_* ≤ 1. When *P_j_* = 1/*m*!, *H_p_*(*m*) reaches the maximum value ln(*m*!).

As concluded from the above procedure, the PE is an appropriate tool for measuring the randomness of a time series. That is, if *H_p_* is small, the time series tends to be regular. Conversely, the time series tends to be random.

### 3.2. Multiscale Permutation Entropy

Multiscale permutation entropy (MPE) is an improved method based on PE. The basic idea is to calculate permutation entropy at multiple time scales. Given a discrete time series, {*x*(*i*), *i* = 1, 2, …, *N*}, the MPE can be calculated as follows [37]:

(1) Construct multiple coarse-grained time series. By averaging the data points within non-overlapping windows of increasing length *s*, time series {*x*(*i*), *i* = 1, 2, …, *N*} can be divided into several coarse-grained time series as follows:

(11) $$y_{j}^{\left(s\right)}=\frac{1}{s}\sum_{i=(j-1)s+1}^{js}x_{i},j=1,2,\cdots ,\frac{N}{s},$$

where s represents a scale factor with 1 ≤ *j* ≤ *N*/*s*, and *y_j_*^(*s*)^ represents the coarse-grained time series. When scale *s* = 1, *y_j_*^(*1*)^ is simply the original time series. 

(2) Calculate permutation entropy at each time scale based on Equations (6)–(10). 

As the vibration signals are complex and random when bearings work under bad conditions, single-scale PE cannot reveal hidden fault information effectively, and PE in other scales also has a major contribution to fault diagnosis. Therefore, MPE was employed to analyze the complexity of reconstructed time series in this paper.

## 4. The Proposed Method

Based on the advantages of the feature space construction and MPE technique, the proposed method encompasses the following steps:

(1) Decompose the rolling bearing vibration signals into a set of IMFs using EEMD. 

(2) The IMFs that contain rich information are selected to reconstruct the feature space according to the criteria of the energy measure.

(3) Calculation the MPE of feature space reconstruction, and the obtained MPEs of the first 10 time scales are selected as the final feature vector.

(4) The final feature vectors are utilized to train the SVM-based multi-fault classifier to identify the fault categories and their severity.

**Figure 5:**
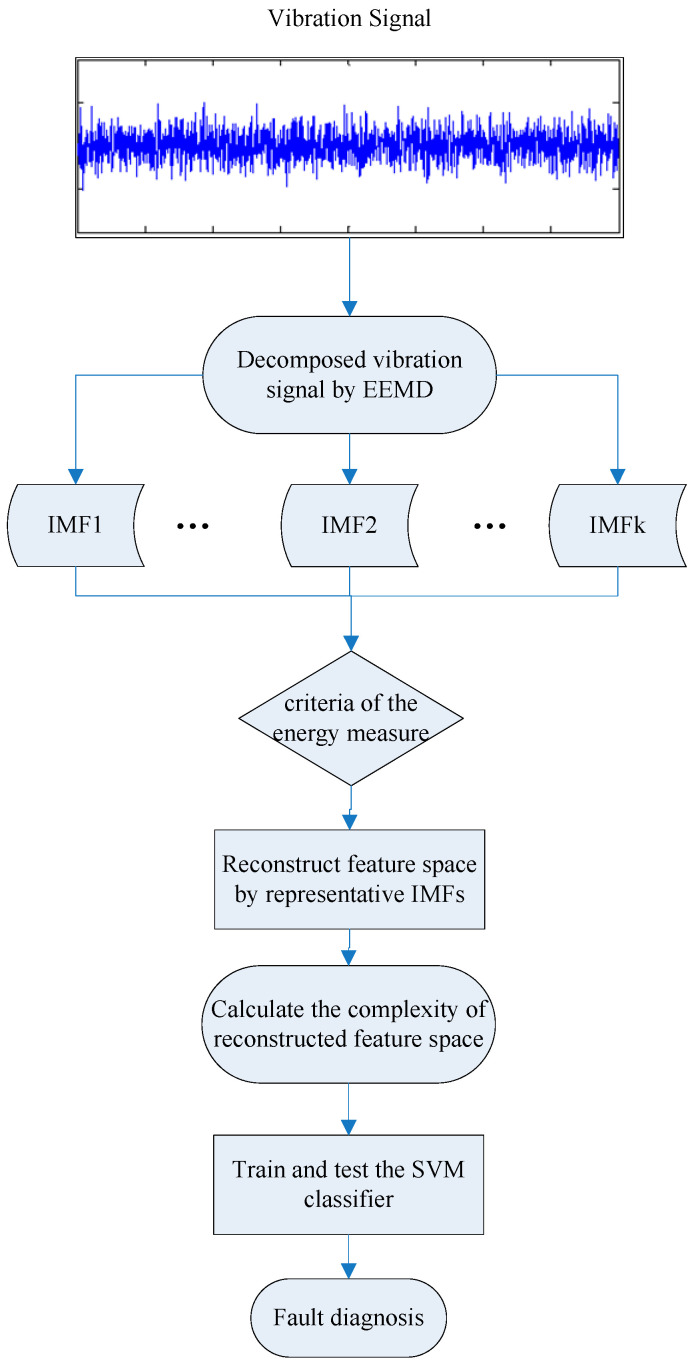
Flow of the presented method.

The flow of the presented method is presented in Figure 5.

## 5. Experimental Results

### 5.1. Experimental Data Description

The experimental data for rolling bearings analyzed in this work were provided by the Case Western Reserve University Bearing Data Center [45]. The test bearing was a drive-end bearing (6205-2RS JEM SKF), which is a deep-groove ball bearing. The motor speed was 1797 rpm, and the sampling frequency was 12 kHz. Vibration signals collected from the normal case (N), ball fault case (B), inner race fault case (IR), and the outer race fault case at the six o’clock position (OR) were selected for this study. The defect sizes of point faults were 0.007 inches, 0.014 inches, and 0.021 inches, respectively. The experimental dataset in this study contained 10 working conditions, including one normal case and nine fault conditions denoted as B007, B014, B021, IR007, IR014, IR021, OR007, OR014, and OR021. Each working condition consisted of 110 samples, and every sample had 1024 data points. Among these samples, 80 samples were randomly selected as training data, and the remaining samples were selected as testing data. The details are given in Table 1.

### 5.2. Results and Analysis

**Figure 6:**
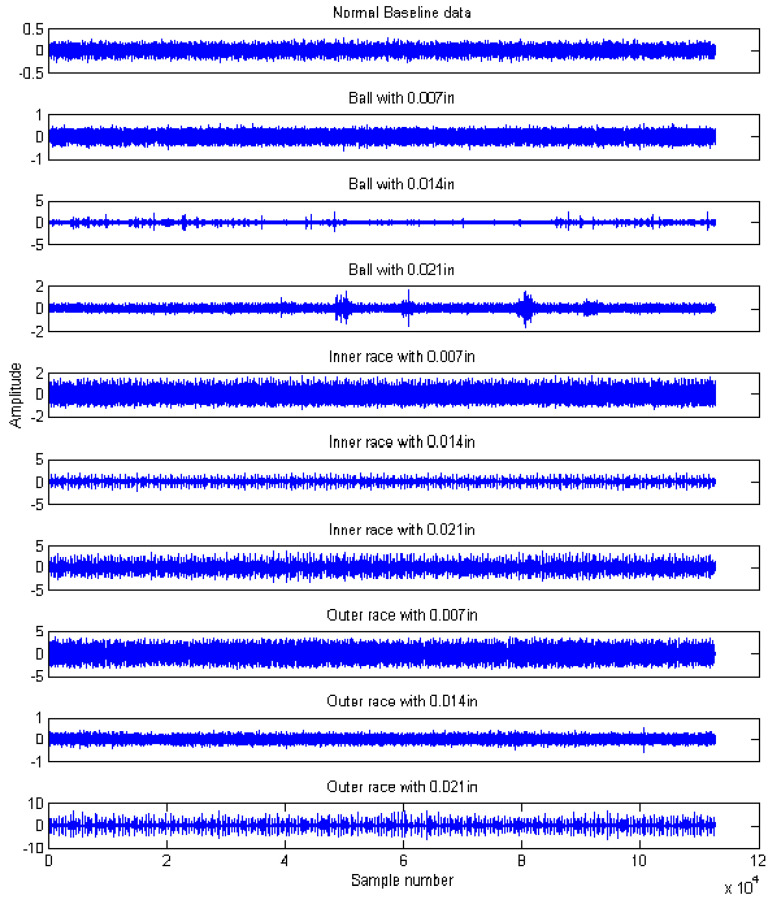
Time domain waveforms of rolling bearings with ten bearing conditions.

**Figure 7:**
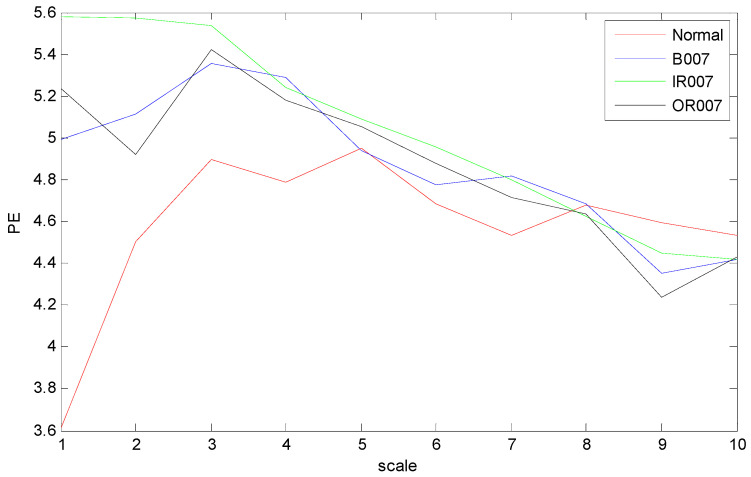
MPE values over 10 scales of bearing vibration signals.

**Figure 8:**
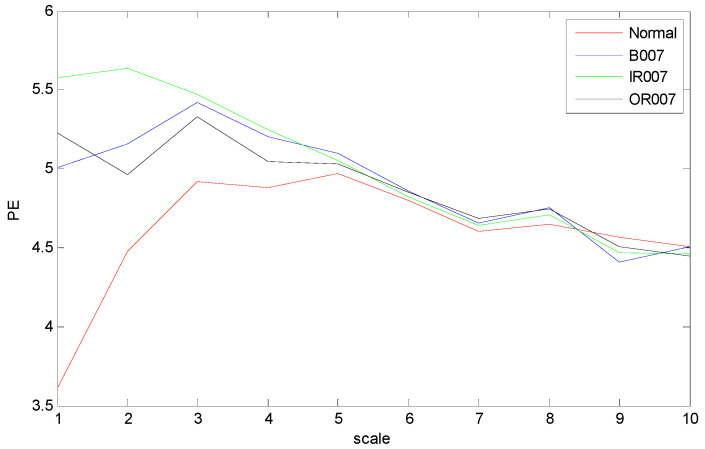
Multiscale permutation entropy (MPE) values over 10 scales of feature space reconstruction.

The time domain waveforms of rolling bearings with the 10 working conditions are shown in Figure 6; it should be noted that it is unreliable to identify different bearing conditions from time domain waveforms. Thus, MPE was used as a nonlinear dynamic technique for bearing fault diagnosis. Figure 7 shows the MPE values of four bearing conditions over 10 scales. From Figure 7, we can find that four kinds of faults could be roughly identified from the different PE values. Among the four categories, the PE values of normal rolling bearings tended to be smaller than the other faults. Furthermore, the fault information in other scales also played an important role for classifying different faults, which implied the importance and feasibility of MPE. However, it appears that the difference in PE values between other faults changed little, such as between the ball fault and outer fault. Meanwhile, the four MPE values crossed each other multiple times and tended to be irregular, which is not conducive to distinguishing faults. Figure 8 presents the MPE results after reconstructing the feature space with FSRE. It is obvious that the different fault types were readily distinguished, and the four MPE value curves were less crossed and overlapped over different scales. This improved the precision and reliability of fault classification.

In this study, three groups of experiments were performed based on the sample data. Group 1 contained one normal case and three fault types with the same defect size of 0.007 inches: B007, OR007, and IR007. Group 2 contained one normal case and three fault types with two defect sizes: B007, B021, IR007, IR021, OR007, and OR021. Group 3 contained all 10 working conditions. The details are presented in Table 2. In the experiments, each sample signal was firstly decomposed by EEMD with the ensemble number *N* = 100, and the amplitude of added noise was 0.2. Then, the FSRE method was utilized to select the important IMFs which contained the main fault information. The feature space was constructed using these IMFs. Then, MPE was adopted to measure the complexity of the feature space. According to the literature [46], the embed dimension was set to 6 and the time lay was set to 1. The MPE values of the first ten time scales were selected as the feature vectors. Finally, the 10 fault conditions which contained 1100 samples with 10 dimensions were acquired and considered as inputs for SVM to recognize the fault patterns of rolling bearings. 

**Figure 9:**
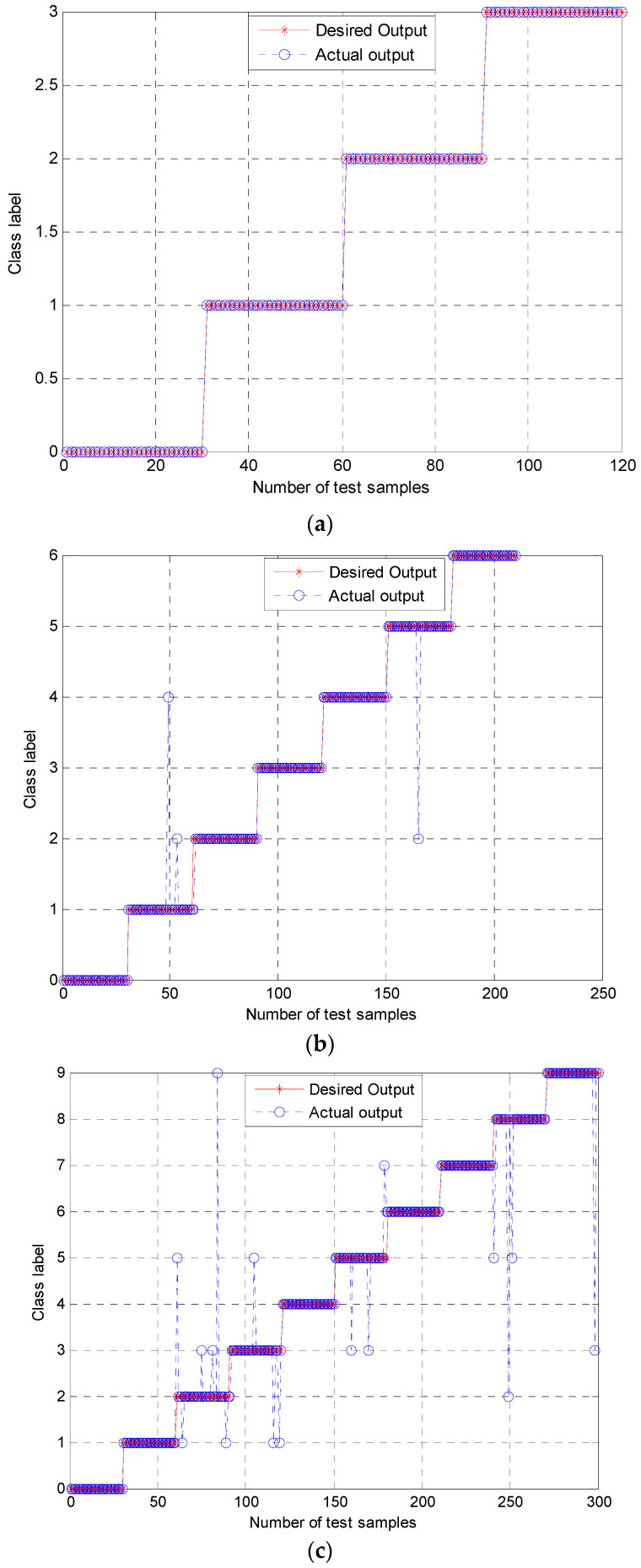
Classification results using the proposed approach: (**a**) Group 1 with four working conditions; (**b**) Group 2 with seven working conditions; (**c**) Group 3 with ten working conditions.

The classification results of the three groups of experiments using the proposed approach are shown in Figure 9. Figure 9a shows that the presented method had a good classification result in Group 1 with a high accuracy of 100%. In Figure 9b, three test samples were misclassified in experimental Group 2, achieving a high accuracy of over 98.57%. Among them, two fault samples with class label 1 (B007) were misclassified into class label 2 (B021) and class label 4 (IR021), and one class label 5 (OR007) was misclassified into class label 2 (B021). The classification result of Group 3 is presented in Figure 9c. It can be observed that sixteen samples were misclassified in a total of 300 samples, with a classification accuracy of 94.67%.

In order to prove the effectiveness of the proposed method, this method was compared with other traditional methods such as bearing fault diagnosis based on single-scale PE, bearing fault diagnosis based on multiscale entropy (MSE), bearing fault diagnosis based on MPE, and bearing fault diagnosis based on intrinsic mode permutation entropy (IMPE) [36]. Each experiment was repeated 10 times, and the average test accuracies of the different methods are reported in Table 3. Group 1 included one normal condition and three fault conditions with different fault types with the same level of fault severity. The experimental analysis was a four-class recognition problem. From Table 3, the average diagnosis accuracy of the single-scale PE method was 92.50%, which was obviously lower than the other methods. It can be found that the single-scale PE method was not sufficient to identify different fault conditions. Compared with the single-scale method, the multiscale methods had good recognition accuracy. Among them, the proposed method and IMPE could achieve a high accuracy of 100%. Group 2 included one normal condition and fault conditions which had different fault types and different levels of fault severity. As the number of classifications increases, the accuracy of classification decreases. This is particularly clear in Group 3, which was a 10-class recognition problem. The classification accuracy of the proposed method was slightly higher than that of the IMPE method, which was significantly higher than that of the other three methods. The reason is that the first three entropy-based methods were directly calculated from the original vibration signal without any feature processing. The IMPE method had a good classification result upon calculating the single PE values of each IMF using EEMD. The presented method firstly eliminated the interference terms or the useless components, such as noise and residue which contribute less to fault diagnosis, using feature space reconstruction, and then calculated the PE values over multiple scales, thereby obtaining a better classification result.

### 5.3. Discussion

The experimental results indicate that the proposed method is a feasible and effective rolling bearing fault diagnosis method. Compared with traditional methods, FSRE had better performance in identifying the rolling bearing faults with different categories and severity. MPE is an effective approach to measure the complexity of time series, such as the vibration signal of bearings in our experiments. Compared with PE and other well-known complexity measures, MPE can better extract and reflect the nonlinear faulty features. The experiments support our assumption that classification performance is better upon calculating MPE values of the vibration signal after feature space reconstruction, compared to directly calculating MPE values of the original vibration signal. This was especially the case when the sample dataset was large with various types of fault, where the proposed framework achieved much higher accuracies than other methods. The classification results of Group 2 and Group 3 agree with this analysis. In this study, the implementation of feature space reconstruction was designed linearly, which is similar to the signal reconstruction method of EMD based on IMFs. Future work can focus on researching more efficient and high-performance reconstruction methods.

## 6. Conclusions

To effectively extract fault features and identify multiple faults (and their severity) of rolling element bearings, in this study, a novel hybrid method was proposed based on feature space reconstruction, MPE, and SVM. As the vibration signals of rolling bearings are complex and change dynamically, the EEMD algorithm was employed to decompose signals into IMFs representing their intrinsic modes. Then, the IMFs which contained rich information were selected to reconstruct the feature space. The results of the simulations confirmed that fault information can be effectively extracted using feature space reconstruction. Additionally, the MPE method was utilized to measure the complexity of reconstructed time series and to generate the fault feature matrix. In order to achieve the fault diagnosis, SVM was applied for fault diagnosis. The results of the three groups of experiments demonstrated the superiority of the proposed method compared to other fault diagnosis methods.

## Figures and Tables

**Table 1:** The details of the 10 conditions.

Working Conditions	Defect Size (inches)	Number of Training Data Points	Number of Testing Data Points	Label of Classification
Normal	0	80	30	0
Ball 1	0.007	80	30	1
Ball 2	0.014	80	30	2
Ball 3	0.021	80	30	3
Inner race 1	0.007	80	30	4
Inner race 2	0.014	80	30	5
Inner race 3	0.021	80	30	6
Outer race 1	0.007	80	30	7
Outer race 2	0.014	80	30	8
Outer race 3	0.021	80	30	9

**Table 2:** The detailed settings of the three groups of experiments.

Group	Fault Label	Label of Classification	Number of Training Data Points	Number of Testing Data Points
1	Normal	0	80	30
	B007	1		
	IR007	2		
	OR007	3		
2	Normal	0	80	30
	B007	1		
	B021	2		
	IR007	3		
	IR021	4		
	OR007	5		
	OR021	6		
3	Normal	0	80	30
	B007	1		
	B014	2		
	B021	3		
	IR007	4		
	IR014	5		
	IR021	6		
	OR007	7		
	OR014	8		
	OR021	9		

**Table 3:** Comparison results of different approaches. PE—permutation entropy; MPE—multiscale permutation entropy; MSE—multiscale sample entropy; IMPE—intrinsic mode permutation entropy.

Approach	Group 1	Group 2	Group 3
PE	92.50%	85.88%	73.25%
MPE	98.33%	94.14%	87.33%
MSE	97.17%	92.46%	84.46%
IMPE	100%	95.85%	91.90%
Proposed method	100%	98.5%	94.7%
